# Hormone stimulation of androgen receptor mediates dynamic changes in DNA methylation patterns at regulatory elements

**DOI:** 10.18632/oncotarget.6471

**Published:** 2015-12-04

**Authors:** Vineet K. Dhiman, Kristopher Attwood, Moray J. Campbell, Dominic J. Smiraglia

**Affiliations:** ^1^ Department of Cancer Genetics, Roswell Park Cancer Institute, Buffalo, NY, USA; ^2^ Department of Biostatistics and Bioinformatics, Roswell Park Cancer Institute, Buffalo, NY, USA; ^3^ Department of Pharmacology and Therapeutics, Roswell Park Cancer Institute, Buffalo, NY, USA

**Keywords:** DNA methylation, androgen receptor, gene transcription, nuclear receptor, androgen regulated target genes, Chromosome Section

## Abstract

DNA methylation is an epigenetic modification that contributes to stable gene silencing by interfering with the ability of transcriptional regulators to bind to DNA. Recent findings have revealed that hormone stimulation of certain nuclear receptors induces rapid, dynamic changes in DNA methylation patterns alongside transcriptional responses at a subset of target loci, over time. However, the ability of androgen receptor (AR) to dynamically regulate gene transcription is relatively under-studied and its role in the regulation of DNA methylation patterns remains to be elucidated. Here we demonstrate in normal prostate cells that hormone stimulated AR activity results in dynamic changes in the transcription rate and DNA methylation patterns at the AR target genes, *TIPARP* and *SGK1*. Time-resolved chromatin immunoprecipitation experiments on the *SGK1* locus reveals dynamic recruitment of AR and RNA Polymerase II, as well as the recruitment of proteins involved in the DNA demethylation process, TET1 and TDG. Furthermore, the presence of DNA methylation at dynamic regions inhibits protein binding and transcriptional activity of *SGK1*. These findings establish AR activity as a contributing factor to the dynamic regulation of DNA methylation patterns at target genes in prostate biology and infer further complexity involved in nuclear receptor mediation of transcriptional regulation.

## INTRODUCTION

Androgens are steroid hormones which exert their biological effects through the androgen receptor, a ligand-dependent nuclear receptor that binds androgen response elements (AREs) in the DNA to regulate gene expression [1]. Nuclear receptors are transcription factors that play important roles in many physiological processes, which include but are not limited to metabolism, development, reproduction and various immune responses [2]. What sets nuclear receptors apart from other transcription factors is that they bind directly to specific lipophilic ligands: steroids, retinoids, thyroid hormones and dietary lipids. Upon ligand binding, nuclear receptors become activated and transcriptionally regulate downstream gene expression pathways by binding to sequence-specific DNA elements and recruiting co-regulatory proteins, chromatin remodeling proteins and components of the basal transcriptional machinery [3].

Early models of transcription illustrated a mechanism that assumed a static chromatin environment, wherein promoter DNA regulatory elements served as stationary platforms to which nuclear receptors and their respective co-regulators bound as stable protein complexes, where they initiated and activated gene transcription [4]. Under this model, it was proposed that these large protein complexes would remain bound to the DNA to continuously regulate transcription for long periods, until stimulus was withdrawn [5]. However, recent studies have contrasted this model, revealing that hormone-dependent transcription is a dynamic signaling process that requires the continuous cyclical recruitment and sequential release of nuclear receptors at DNA response elements [6]. This model has been demonstrated under hormone-stimulated conditions for the glucocorticoid receptor (GR) [7], the estrogen receptor α (ERα) [8], the vitamin D receptor (VDR) [9, 10], and the retinoid X receptor (RXR) [9]. The dynamic recruitment of these nuclear receptors to their respective DNA response elements has been reported to occur in phase with gene transcription. An in-depth analysis of ERα binding to the *TFF1/pS2* promoter revealed that alongside the dynamic recruitment of ERα, co-factors and chromatin modifiers, such as histone acetyltransferases (HATs) and histone deacetyltransferases (HDACs), were also recruited in a similar fashion [8]. Similarly, the androgen receptor (AR) has also been reported to be dynamically recruited to targets genes to mediate transcription [11]. However, these dynamics have only been reported in prostate cancer cells on the *PSA* locus, and the relevance of dynamic AR signaling to normal prostate biology remains unexplored.

Epigenetic DNA modifications are heritable marks that are required for correct gene expression and for the compartmentalization of the genome into euchromatic and heterochromatic regions [12]. When used in many diverse and complex combinations, these modifications regulate cell reprogramming and dictate cell fate decisions during stages of development and differentiation. Additionally, epigenetic DNA modifications are known to be largely responsible for dictating chromatin remodeling structures in order to regulate gene expression profiles.

An important example of an epigenetic modification is DNA methylation. DNA methylation is a chemical modification that involves the covalent addition of a methyl group to the fifth carbon of a cytosine residue and typically occurs within a CpG dinucleotide context in adult somatic cells [13]. Not only is DNA methylation vital for mammalian development and adult homeostasis, but it is also a central mechanism of epigenetic regulation in eukaryotic cells [13-15]. DNA methylation controls important biological functions, such as inactivation of the X chromosome, genomic imprinting and the regulation of gene expression [16]. DNA methylation is associated with stable gene silencing, either through the interference of transcription factor binding to the DNA or through the recruitment of several repressor proteins that bind to sites containing methylated DNA, consequently creating a repressive transcriptional environment.

While the DNA methyltransferase family of proteins (DNMTs) has been well-known to catalyze the addition of a methyl group to the number five carbon in a CpG dinucleotide [14], it was not until the discovery of the role of the ten eleven translocation (TET) family of proteins that the mechanism of DNA demethylation became fully understood. These studies have shown that the TET enzymes are able to successively oxidize 5-methylcytosine to 5-hydroxylmethylcytosine, 5-formylcytosine and 5-carboxylcytsonine [17, 18]. Thymine DNA glycosylase (TDG) recognizes these oxidized bases and excises them, which paves the way for DNA repair mechanisms to replace the base with an unmethylated cytosine [18]. These observations have led to a renewed focus on the dynamics of DNA methylation in all contexts of cell function. Many of these studies have focused on comparing global methylation dynamics that dictate embryonic stem cells before and after lineage commitment and differentiation [19]. These cell states are typically differentiated on a timeline of days to weeks [20]. However, locus-specific dynamic methylation on the order of minutes remains largely unexplored. While reports have indicated that DNA methylation is a dynamic mark that is associated with ERα signaling in the context of a transcriptional response [21, 22], a role for dynamic DNA methylation in relation to androgen receptor (AR) signaling, has not been investigated to date.

Here we demonstrate that hormone stimulation of AR leads to dynamic patterns in the transcriptional rate of AR target genes in normal, non-transformed prostate epithelial cells. This occurs along with dynamic changes in the DNA methylation patterns at androgen response elements (AREs) within these genes. Additionally, we show that AR, TET1 and TDG are all seen to be dynamically co-recruited to these regions. These data establish a central role for the hormonal stimulation of AR in the regulation of the DNA methylation pattern of AR target genes, alongside the more elucidated role of AR as a transcriptional regulator.

## RESULTS

### HPr1-AR cells are a normal, prostate cell line that expresses AR and semi-differentiates in response to DHT

**Figure 1:**
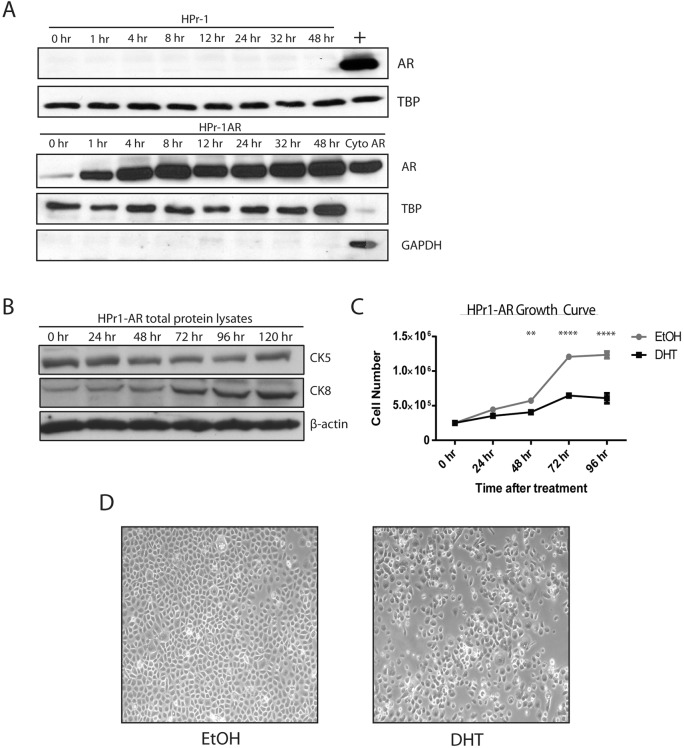
Characterization of short-term androgen treatment of HPr1-AR cell lines **A.** HPr1 (upper panel) and HPr1-AR cells (lower panel) were treated with 10 nM DHT. Nuclear lysates were harvested at indicated time points for western blot analysis for AR and TBP. Positive control (+) represents HPr1-AR nuclear lysate stimulated with DHT for 48 h. Cyto AR represents the cytoplasmic fraction for AR, stimulated with DHT for 48 h. **B.** Cells were treated with 10 nM DHT are harvested every 24 h for total protein. Lysates were used in western blot analysis to determine differentiation potential of HPr1-AR cells. **C.** Cellular proliferation was determined using trypan blue staining. HPr1-AR cells were treated with 10 nM DHT and counted every 24 h. **D.** Microscopy representation of HPr1-AR cells after 48 h of EtOH vehicle or 10 nM DHT treatment. (Student T-test, **p < 0.01, ****p < 0.0001).

To study the association between androgen receptor (AR) signaling and DNA methylation patterns in normal prostate biology, we used the Human Prostate-1 (HPr1) cell line that stably expresses exogenous AR under control of the CMV promoter (HPr1-AR) [23]. These cells constitutively express AR protein in the cytoplasm but demonstrate nuclear accumulation of AR and differentiate in response to long term androgen-exposure [23]. However, their short-term response has not been characterized. We observed that short-term treatment with 10 nM dihydrotestosterone (DHT) for as little as 1 h resulted in an increase in nuclear AR localization, as demonstrated by western blot analysis of nuclear lysates (Figure 1A, lower panel). As expected, AR protein expression is completely absent in the parental cell line, HPr1 (Figure 1A, upper panel). We next investigated whether HPr1-AR cells differentiated in response to short-term DHT treatment by measuring the expression of differentiation cytokeratin markers. A decrease in the basal marker CK5, paired with a simultaneous increase in the luminal marker CK8, shows that the HPr1-AR cells semi-differentiate toward a more luminal-like phenotype when treated with DHT (Figure 1B, 1D). Additionally, a significant decrease in the growth rate of the cells after 48 h of DHT treatment was observed (Figure 1C). These results indicate that the HPr1-AR cells behave similar under short-term androgen treatment as they do under long-term treatment [23] and provide an ideal model to study the AR signaling axis, as a non-malignant, immortalized prostate cell line that expresses AR.

**Figure 2:**
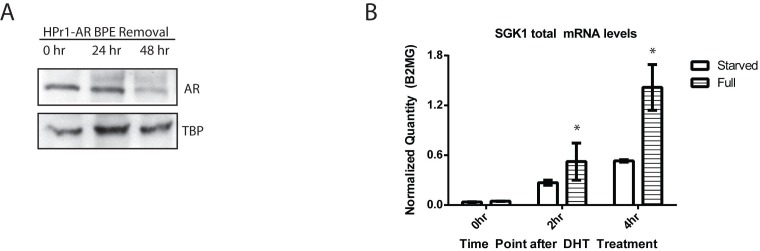
Bovine pituitary extract removal reduces nuclear AR levels and results in more reproducible transcript levels **A.** Bovine pituitary extract (BPE) was removed from the growth media and nuclear lysates were collected every 24 h. **B.** HPr1-AR cells were maintained in either full or BPE-removed media conditions and then treated with EtOH vehicle or 10 nM DHT for 0 h, 2 h or 4 h. Total mRNA expression was analyzed via qRT-PCR. Error bars of indicative of standard error. (Student T-test, *p < 0.05).

Studies of transcriptional dynamics often begin with extraordinary efforts to synchronize cells such as 72 hours of serum starvation followed by a short treatment with the RNA polymerase inhibitor α-amanitin to turn off transcription in all cells. This is followed by washing out the transcription inhibitor and adding the stimulus. While this is highly effective at synchronizing transcriptional response to the stimulus, thereby allowing for detection of coordinated dynamics, it places the genome in an artificial basal state that might influence the basal DNA methylation we want to study. Such an artificial basal state might then exaggerate or subdue any potential dynamic changes to methylation in a short time frame. Omitting synchronization of the cells other than the addition of ligand (androgen) allowed for detection of dynamic transcription and DNA methylation in individual replicates, however there was too much variation in biological triplicates to be confident of the data (data not shown). Therefore, we explored alternate methods of cell population synchronization that relied on growth factor withdrawal. The growth media for HPr1-AR cells contains bovine pituitary extract (BPE) that may be a source of potential hormone that can activate AR. BPE starvation of the cells reduced basal nuclear AR levels (starved media condition) in the absence of DHT. Western blot analysis of nuclear lysates in Figure 2A demonstrated that after 48 h of BPE withdrawal, nuclear AR levels were decreased.

To determine if this method of synchronization resulted in a robust and consistent transcriptional response to the addition of DHT, total mRNA levels of target genes were measured in cells that were either grown in BPE-containing or BPE-starved media for 48 h, and then treated with DHT. While the magnitude of the transcriptional response to the addition of ligand at 2 h and 4 h was muted in the starved cells compared to the cells grown in full media, the biological triplicates were far more consistent with tighter error bars in the BPE-starved cells (Figure 2B). We determined that the reduction in signal amplitude was an acceptable trade-off to facilitate our goal of observing dynamics that were highly consistent across replicates.

### Androgen-induced dynamics in transcriptional output occurs alongside dynamic changes in the DNA methylation patterns at gene regulatory elements

**Figure 3:**
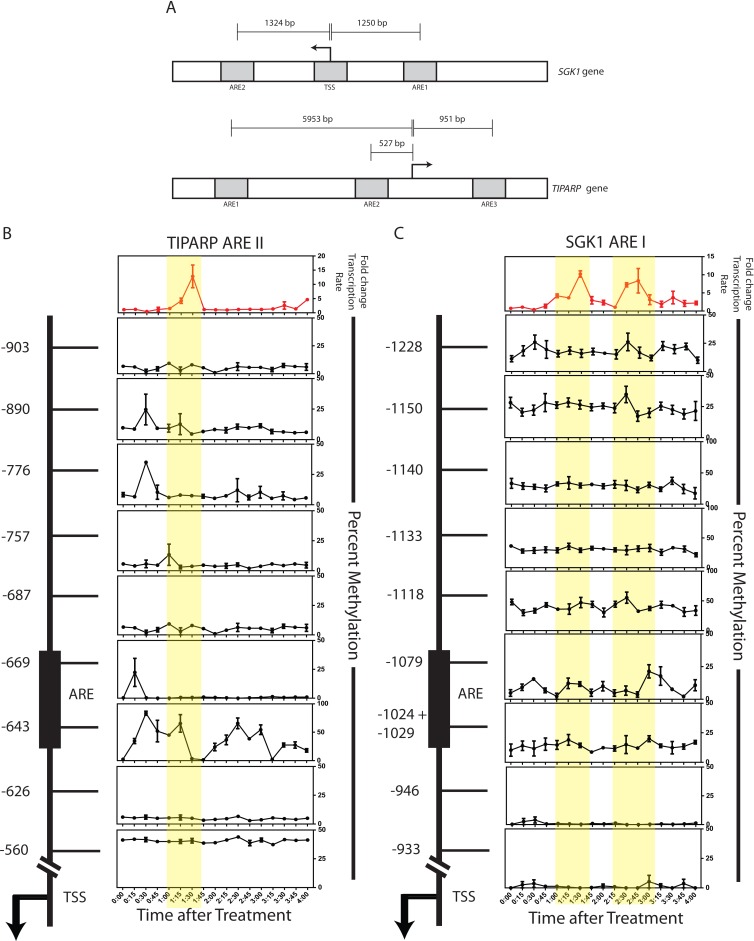
Androgen-induced transcriptional output correlates with dynamic changes in the DNA methylation patterns of gene regulatory elements **A.** Schematic representation of the *SGK1* (upper panel) and *TIPARP* (lower panel) loci. Outlined in gray boxes are the ARE regions interrogated for methylation analysis. **B.**-**C.** HPr-1AR cells were treated with 10nM DHT and harvested for RNA and DNA, from the same cell pellet, at the indicated time points. Transcription data is reflective of normalized quantity of nascent gene expression. Expression data was normalized to β-microglobulin and representative of the mean of three biological replicates. CpG sites in the *TIPARP* ARE II and *SGK1* ARE I regions were interrogated for methylation. Each row represents a single CpG site, where each CpG site is labeled in respect to its distance in base pairs from the TSS (as noted by the arrow). The black box represents the confirmed androgen response element (ARE) from previously published ChIP-chip studies. [24] All methylation data points are representative of the mean of three biological replicates or best two out of three replicates. Error bars of indicative of standard error.

To assess the correlation between the transcriptional rate of AR target genes and their respective DNA methylation patterns, both RNA and DNA were collected from the same cell population every 15 min after DHT treatment over a 4 hour window. All experiments were performed as biological triplicates and the transcription rate is represented as fold change over the vehicle control at each of the 17 time points. We interrogated two AR target genes, *TIPARP* (TCDD-Inducible Poly(ADP-Ribose) Polymerase) and *SGK1* (serum/glucocorticoid regulated kinase 1), which have been reported previously to be up-regulated upon hormone treatment in HPr1-AR cells [24]. Nascently transcribed RNA was isolated to measure the transcription rate of these two genes over the timeline. As shown in Figure 3B, *TIPARP* has one transcription rate peak that occurs between 1:15 h and 1:45 h after DHT treatment and then another peak of transcriptional activity that begins 4 h after treatment. *SGK1* has two distinct transcription rate peaks that occur between 0:45 h through 1:45 h and between 2:15 h through 3:00 h (Figure 3B-3C, top graphs). These data establish that DHT stimulation of AR leads to dynamic transcription rates of AR target genes.

We used MassARRAY EpiTYPER to quantitate the methylation status of CpG dinuceotides located near or at AREs of the TIPARP and SGK1 loci to determine if the regions displayed changes in DNA methylation in relation to oscillations in transcription rate over time. Figure 2A outlines the regions that were interrogated. We observed methylation oscillations at CpG sites at or near the AREs in response to DHT treatment. Specifically, DNA demethylation was observed at ARE II of *TIPARP* between 1:15 h and 1:45 h after DHT treatment at CpG −643, which correlates with an increase in the transcriptional rate (Figure 3B). This is preceded by drops in methylation levels at CpGs −669, −776, and −890 at the 0:30 and 0:45 time points. Furthermore, when the transcriptional rate decreases after the 1:45 time point, methylation returns to CpG −643. Spearman correlation analysis reveals that CpG sites −643 and −890 negatively correlate significantly with the transcription rate 15 minutes later (Table 1.1). This suggests that the methylation status at these sites may be predictive of the expression pattern of *TIPARP.* Interestingly, CpG −669 correlates positively with transcription rate 15 minutes later.

**Table 1.1:** Spearman's Correlation Coefficient for *TIPARP* transcription rate vs. methylation

CpG Site	Correlation Coefficient	*P*-value
−560	−0.344	0.192
−626	−0.167	0.535
−643	−0.612	*0.012*
−669	0.539	*0.031*
−687	0.134	0.621
−757	0.073	0.789
−779	−0.376	0.151
−890	−0.549	*0.028*
−903	0.134	0.621

Spearman's correlation coefficients were determined by comparing the expression values with a lag of 15 minutes prior to the methylation values at a given time point.

We further observed dynamic changes in DNA methylation at the ARE I region of *SGK1* at CpG sites −1024/−1029, −1079, −1118, −1150 and −1228 (Figure 3C). These dynamic regions are in contrast to CpG sites −933, −946, −1133 and −1140, which do not exhibit dynamics. DNA demethylation occurs from 0:30 h to 1:00 h and also from 2:00 h to 2:45 h at CpG −1079, in phase with an increase in the transcriptional rate during each time window (Figure 3C). Similar statistical analysis on the predictive measure of the methylation on the transcription rate revealed no significant correlations at any of the tested CpG sites (data not shown). Statistical analysis on the change in methylation compared to the change in the transcription rate also revealed no significant correlations (Table 1.2). However, it is noteworthy that most of the CpG sites in the ARE I region of *SGK1* have a negative correlation value in relation to the transcription rate (Table 1.2). These analyses suggest that the regulation at *SGK1* may be a more complex mechanism than at *TIPARP*.

**Table 1.2:** Spearman's Correlation Coefficient for *SGK1* transcription rate *vs*. methylation

CpG Site	Correlation Coefficient	*P*-value
−933	0.637	*0.060*
−946	−0.500	0.207
−1024/−1029	0.357	0.385
−1079	−0.286	0.493
−1118	0.286	0.493
−1133	−0.452	0.260
−1140	−0.262	0.531
−1150	−0.048	0.910
−1228	0.286	0.493

Spearman's correlation coefficients were determined by comparing the change in SGK1 transcription rate to the change in methylation values at each time point.

The ARE I region for *TIPARP* and the ARE II and TSS (transcription start site) regions for *SGK1* did not show any methylation dynamics at the time points and conditions used in our study (Supplementary Figure 1). Collectively, these data demonstrate that context-dependent, dynamic DNA methylation occurs at select AREs in response to androgen stimulation, suggesting a link between AR signaling and DNA methylation.

### Dynamic recruitment of AR, TDG and TET1 is observed at the SGK1 ARE I

**Figure 4:**
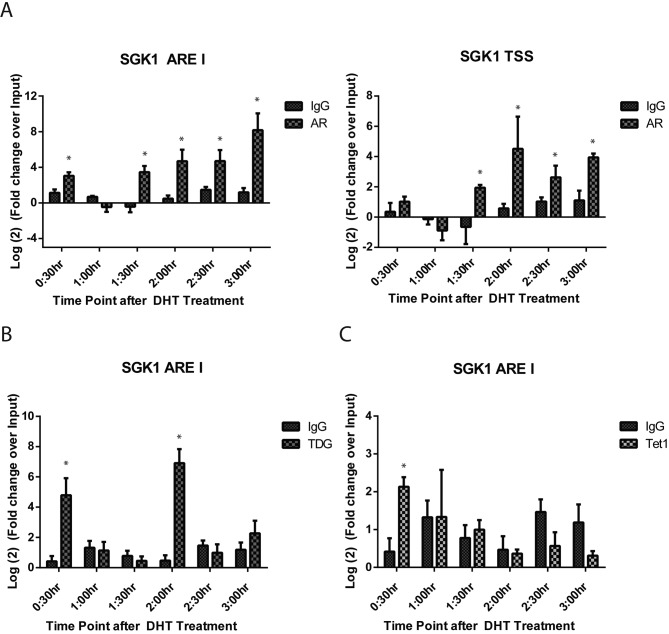
Dynamic recruitment of AR, TDG and TET1 is observed at the *SGK1* ARE I HPr-1AR cells were treated with EtOH vehicle or 10 nM DHT for indicated time points. Regulatory element occupancy was determined using antibodies against **A.** AR, **B.** TDG and **C.** TET1 and normalized and presented as fold change over input. ChIP signal was measured using qRT-PCR with primers specific to the interrogated region. All data points are representative of biological triplicates. Error bars are indicative of standard error. (Student *T*-test, *p < 0.05).

We next used (chromatin immunoprecipitation) ChIP to examine the molecular details of protein recruitment to the *SGK1* ARE I region. Treatment of HPr1-AR cells with DHT resulted in dynamic recruitment of AR to this region; specifically, significant AR recruitment was observed at 0:30 h, then lost at 1:00 h, followed again by occupancy of AR at 1:30 h (Figure 4A). Interestingly, we observed persistent recruitment of AR at each time point following 1:30 h of DHT treatment. Analysis of the *SGK1* TSS region revealed a similar pattern of AR recruitment. Similar patterns of dynamic recruitment were also observed for RNA polymerase II in the first 1:30 h after DHT treatment (Supplementary Figure 2). Together, these ChIP data for AR and RNA Pol II demonstrate that both proteins are dynamically recruited alongside one another to mediate gene transcription in response to DHT.

DNA demethylation has been attributed to the function of TET1 and TDG proteins. Therefore, ChIP for TET1 and TDG at the *SGK1* ARE I region was performed. There was significant recruitment of TET1 and TDG to ARE I at 0:30 h after DHT treatment (Figure 4B-4C). While there was no other observed recruitment of TET1 at other time points, we were able to detect a second cycle of TDG occupancy at 2:00 h. The recruitment of TET1 and TDG at the 30 minute time point is 30 minutes prior to the first increase in transcriptional rate, and 15-30 minutes preceding demethylation that was observed for CpG sites −1079 and −1288. The recruitment of TDG at the 2:00 time point precedes a transcriptional rate peak by 30 minutes and demethylation events at positions −1288, −1150, −1140, and −1079 by 15-30 minutes.

### Methylation of CpG sites at the SGK1 ARE I inhibits protein binding and androgen responsive transcription

**Figure 5:**
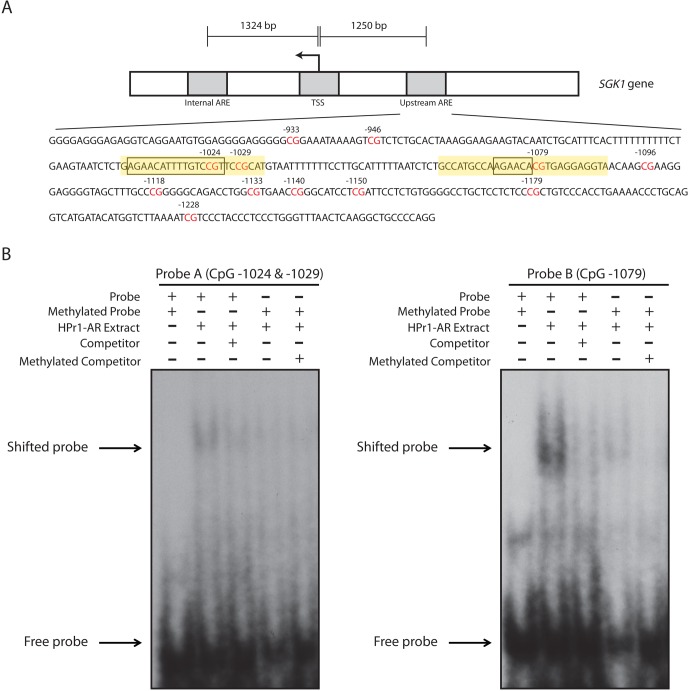
Methylation of dynamic CpG sites at the *SGK1* ARE I inhibit the binding of protein to DNA **A.** Schematic representation of the *SGK1* locus. An in-depth sequence analysis of the ARE I region illustrates 12 different CpG sites (highlighted in red). The ARE I region contains two AR binding sequences (as boxed), where one binding site is representative of a full consensus sequence and the second site is representative of a half-site. Designed DNA probes for EMSA analysis are highlighted in yellow. Probe A consists of CpG sites −1024 and −1029. Probe B consists of CpG site −1079. **B.** DNA probes were either unmethylated or methylated, radiolabeled, and were incubated with DHT-stimulated HPr1-AR nuclear extract, an unlabeled competitor probe and/or and unlabeled methylated competitor probe.

We next sought to investigate the significance of methylation at the CpG positions showing dynamic responses. We hypothesized that methylation of these CpGs would prevent the binding of transcriptional regulatory proteins. We utilized Electromobility Shift Assays (EMSA) to interrogate protein-DNA binding to DNA probes that overlapped the AR consensus sequences and the dynamic CpGs that were observed in the *SGK1* ARE I region. Because there were two AR consensus binding sites in this region, we created two different probes that covered both of these sequences: one probe (Probe A, 24 nts in size) that covers CpG sites −1024 and −1029 and a second probe (Probe B, 27 nts in size) that covered CpG site −1079 (Figure 5A). When we incubated our DNA probes with DHT-stimulated HPr1-AR nuclear extract, we observed a shift that indicated binding of protein to both probes, with clearly strong binding to Probe B. All lanes included 200x molar excess of non-specific competitor poly dI:dC to block non-specific interactions. The gel shift was effectively competed out by the introduction of a specific competitor, the unlabeled probe (Figure 5B) at 200x molar excess. These results demonstrate specific nuclear protein binding to both probes. To determine the effect that methylation has on protein binding, we used SssI methylase to generate fully methylated versions of both probes. Methylation resulted in a lack of gel shift for both probes indicating that methylation interferes with protein binding (Figure 5B). Note that despite only having 2 CpG sites in Probe A (−1024 and −1029) and 1 CpG site in Probe B (−1079), their methylation was sufficient to greatly inhibit protein binding. This suggests that the dynamic demethylation of these CpG sites in response to DHT treatment is likely a prerequisite for the recruitment of DNA-binding regulatory factors that contribute to the transcriptional response.

**Figure 6:**
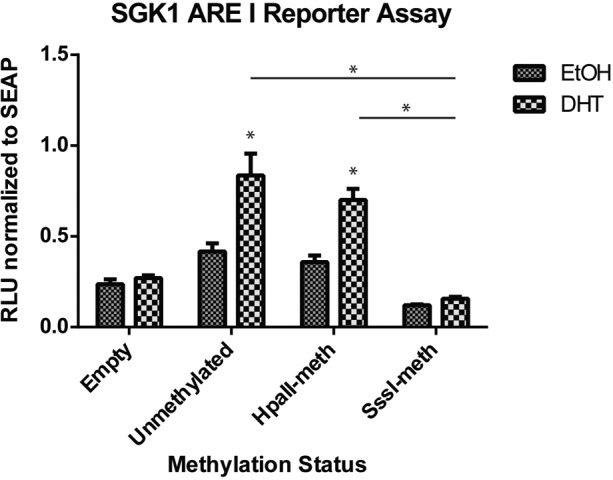
Methylation of the *SGK1* ARE I region inhibits transcriptional activity The *SGK1* ARE I region was cloned into the pCpGfree-promoter vector. Unmethylated, partially methylated (HpaII) and completely methylated (SssI) versions of the vector were transfected into HPr1-AR cells. Cells were treated with EtOH vehicle or 10 nM DHT for 48 h and assayed for Lucia (RLU) and secreted alkaline phosphatase (SEAP) expression. All data points represent three biological replicates. (Student *T*-test, *p < 0.05).

To assess the biological function of methylation of ARE I of the *SGK1* locus, we inserted a 450 bp fragment containing the ARE I region into the pCpGfree-promoter-Lucia reporter vector upstream of the hEF1 promoter. The CpG free nature of this vector allows for artificial methylation of only the inserted DNA by incubation with methyltransferases. We created both partially (2 of 12 CpG sites, −1118 and −1140) and fully (12 of 12 CpG sites) methylated versions of the ARE I region using HpaII or SssI methylase enzymes, respectively, to test the effect of DNA methylation on promoter activity. Upon transfection and treatment with DHT, the unmethylated vector significantly induced transcriptional activity in HPr1-AR cells, confirming the androgen responsiveness of this element (Figure 6). Partial methylation of ARE I had no effect on androgen response. However, the fully methylated *SGK1* ARE I resulted in a complete block in androgen responsiveness. These data establish the ARE I within the *SGK1* gene as a positive regulator of *SGK1* transcription, with putative enhancer activity. Further, our findings indicate that methylation of this region, which displays dynamic DNA methylation changes in response to androgen treatment, plays an inhibitory role in *SGK1* transcription.

## DISCUSSION

The AR signaling axis governs and influences pro-differentiation transcriptional programs in normal prostate biology. In the current study, we have demonstrated that DHT stimulation of AR results in dynamic changes in the DNA methylation patterns at biologically active AREs. At select CpG sites, this dynamic methylation pattern occurs inversely in phase with dynamic changes of the transcriptional rate of target genes. These data show that dynamic changes in DNA methylation also play a role in transcriptional regulation. These data correlate with the already-established dynamic histone modification patterns that are observed with the recruitment pioneer factors, nuclear receptors, nuclear receptor co-factors and the transcriptional machinery, providing evidence that DNA methylation also plays a dynamic role in the complex process of transcriptional activation.

The CpG sites, which coordinate most closely with the transcriptional pattern, are CpG sites that are located near or within AREs. Notably, there are CpG sites that surround the AREs that show little or no methylation dynamics, suggesting that only select CpG may play a role in transcriptional regulation. Most of the other regions that did not show dynamics were either completely methylated or unmethylated across the interrogated region. The TSS region of *SGK1* sits within a CpG island, which was almost uniformly unmethylated at all CpG positions across all time points. These observations fit with previously published data that show regions where dynamics occur are typically moderately methylated (anywhere from 10-50% methylated) and in regions of relatively low density of CpG [25, 26]. These findings suggest that the selective methylation dynamics that occur specifically at the lower CpG density AREs are essential for androgen-dependent transcriptional activation.

Despite observed methylation dynamics, most CpG sites not located directly at the AREs do not show significant correlation between the methylation dynamics and the transcriptional rate, suggesting that these CpG sites do not play direct roles in regulation transcription, but may influence chromatin remodeling, the binding of other transcription factors, or other processes that may be prerequisites to initiating or terminating androgen-dependent transcription. Support for this hypothesis stems from previous reports which demonstrated that transcription by nuclear receptors appears to be associated with dynamic changes in DNA methylation [21, 22, 27-29]. When we interrogated ENCODE ChIP-seq data, we observed a plethora of other TFs that binds near to the *SGK1* ARE I and the *TIPARP* ARE II (data not shown), which may explain the observed dynamics at these other CpG sites. Furthermore, the presence of methylation dynamics at distal regulatory regions as opposed to promoter regions is consistent with data that links DNA hypomethylation at enhancer regions to transcription factor binding and enhancer activity [25, 26, 30]. These observations have been shown in both mouse and human cells.

Whether or not AR itself is responsible for dictating methylation patterns during transcriptional control remains to be determined; however, the link between androgen stimulation, selective transient DNA demethylation, and the recruitment of AR, TET1, and TDG is clear. Previous reports have linked DNMT3A/3B to the process of DNA demethylation when interacting with TDG *in vitro* [22]. TDG is observed to inhibit the methylation activity of DNMT3A while DNMT3A stimulates the glycosylase activity of TDG. However, there have been follow up studies which suggest that the sequential actions of DNMT-mediated deamination and base excision repair are not enough to achieve hormone-induced active DNA demethylation [31]. The discovery of the role the TET family of proteins in the DNA demethylation pathway has brought these proteins to the forefront of the demethylation process. Studies have shown that TET1 has been found to be associated with promoter regions and regulatory elements [32]. In line with these observations, we have found TET1 to be present at the *SGK1* ARE I. This supports the notion that not only is TET1 involved in DNA demethylation during cell lineage decisions and development, but also may play a role in the transient transcriptional response of hormone-inducible genes. The presence of multiple cycles of TDG association with the *SGK1* ARE I in ChIP assays is consistent with previous published data that have observed TDG recruitment [22], and suggests TDG is dynamically recruited to mediate transcription. Of note is the observation that NCOA3, a common co-activator of the AR [33], binds TDG [34]. Furthermore, interference with the ability of NCOA3 to bind TDG decreased the activity of AR signaling. This supports a central role that AR may play in the temporal recruitment of a putative protein complex that consists of the enzymes involved in the DNA demethylation process to initiate chromatin-remodeling processes for transcription.

While the broad, inhibitory role that DNA methylation plays in the regulation of transcription is an accepted function of the epigenetic mark, the observation that methylation patterns are dynamically changing to regulate transcription in lineage committed cells is still poorly understood. The current study reports for the first time that hormone stimulation of AR has been linked to these observations, and as a result, introduces potential new pathways to consider for AR target gene regulation. Consistent with published data, the methylation dynamics that we observe occur at low density CpG regions [22, 25], which may be more likely to be involved in dynamic gene regulation. Even for the TIPARP region showing methylation dynamics, which is near the end of a CpG island, the methylation is very focal with only few specific CpGs showing methylation dynamics, therefore limiting the methylation density within the region. This is in contrast to regions of the genome of high CpG density, where high level methylation of these regions are typically associated with stable gene silencing and heterochromantinization [35]. Aberrant DNA methylation and heterochromantinization at gene promoters, which are commonly found to contain high CpG density regions [15], are known epigenetic hallmarks of cancer. As such, understanding the importance of DNA methylation in these contexts in normal biology may shed light on how DNA methylation patterns become distorted in cancer.

## MATERIALS AND METHODS

### Cell culture

Human Prostate 1 (HPr-1) cells were grown in Keratinocyte-Serum Free Medium (SFM) (1x) (Life Technologies, Cat #17005-042), supplemented with bovine pituitary extract (BPE) and 2 μg/ml epidermal growth factor-1 (EGF1). The HPr-1AR cells which stably overexpress exogenous AR [23] were grown and maintained in the same conditions, along with 2 μg/ml puromycin. For starvation conditions, HPr-1AR cells were grown in Keratincyte-SFM without BPE or EGF1 for 48 h. For experimental conditions, HPr-1 and HPr-1AR cells were treated with 10 nM dihydrotestosterone (DHT) (Sigma, Cat# A8380-1G) or ethanol (EtOH) as a vehicle.

### RNA and DNA isolation

RNA and DNA were isolated from the same cell pellet. RNA was isolated using Trizol^®^ (Life Technologies, Cat #15596-026), as per the manufacturer's instructions. RNA was resuspended in molecular grade water and stored at −80°C.

DNA was isolated using Trizol^®^ reagent, with a modified protocol. After the aqueous RNA-containing phase was removed, all samples were spun down at 12,000 g for 5 min at 4°C. 750 μl of Back Extraction Buffer (BEB) (4 M guanidine thiocyanate, 50 mM NaCi, 1 M Tris base) was added to all samples and then mixed for 10 min at room temperature (RT). Samples were spun down at 12,000 g for 30 min at RT. The upper aqueous phase was retrieved, to which 400 μl of isopropanol was added for each 1 ml of Trizol^®^ reagent. Samples were mixed and incubated at RT for 5 min and then spun for 12,000 g for 15 min at 4°C. The resulting DNA pellet was washed with 500 μl of 70% EtOH. Samples were spun again at 12,000 g for 15 min at 4°C; the supernatant was removed and then the DNA pellet was dissolved in 400 μl of 1x TE Buffer.

To further purify the DNA, 400 μl of phenol chloroform isoamylalcohol (PCI) (25:24:1) was added to each sample. Samples were mixed for 10 min at RT via rotation, and then spun down at 12,000 g for 15 min at RT. A second PCI extraction was performed afterwards. The upper aqueous phase was retrieved. 20 μl of 3M sodium acetate (NaOAc) pH 5.2, 2 μl of 1 mg/ml glycogen and 2.5x volumes of 95% cold EtOH was added to precipitate the DNA. Samples were spun at 13,000 rpm for 15 min at 4°C. The resulting DNA pellets were then washed with 100 μl of 70% cold EtOH and spun down again at 13,000 rpm for 15 mins at 4°C. DNA was resuspended in 50 μl of 1x TE buffer and stored at 4°C.

### Nascent RNA capture

HPr-1AR cells were treated with 10 nM DHT or EtOH as a vehicle every 15 min for 4 hrs in biological triplicates. The cells were pulsed with 0.5 mM 5-ethynyl uridine (5-EU) (Jena Biosciences, Cat #CLK-N002-10) for 30 min before harvesting in 1.0 ml Trizol^®^ reagent for RNA isolation. To biotinylate the RNA, a copper catalyzed click reaction was performed. The reaction cocktail was set up as follows: 1.0 μg of RNA, 4.64 μl 10 mM Biotin Azide (Jena Biosciences, Cat #CLK-FA003-1), 6.96 μl click reaction (1 part of 0.1 M Copper Bromide, 2 parts 0.1 M Tris[(1-benzyl-1*H*-1, 2,3-triazol-4-yl)methyl]amine (TBTA) in 3:1 DMSO/t-BuOH). The click reaction cocktail was incubated at 37°C for 3 hours. 0.3 M NaOAc and 1.0 ml 100% cold EtOH was added to precipitate the RNA. Samples were incubated at −80°C overnight. They were then spun down at 12,000 g for 20 min at 4°C. Nascent RNA pellets were washed with 70% cold EtOH and spun at 12,000 g for 5 min at 4°C. Pellets were resuspended in 50 μl of molecular grade water and stored at −20°C until further use.

To capture nascent RNA, the following binding reaction was set up: 2.0 μl RNaseOUT™ recombinant ribonuclease inhibitor (Life Technologies, Cat #10777-019), 1.0 μg biotinylated DNA, 250 μl nucleic acid binding and wash buffer (50 mM Tris-HCl, 150 mM NaCl, 0.05% Tween 20, pH 8.0), and 25 μl streptavidin magnetic beads (Solulink, Cat #M-1002). The capture reaction was then rotated at RT for 1 h. The beads were then washed with nucleic acid binding and wash buffer (rotated for 5 min at RT) 5 times. After the final wash, the beads were resuspended in 50 μl molecular grade water. The First Strand cDNA synthesis kit (Thermo Scientific, Cat #K1622) was used to generate cDNA using the nascently captured RNA bound to the beads as template. The manufacturer's instructions were followed. cDNA was stored at −20°C.

### qRT-PCR

Quantitative PCR reactions were carried out using Sybr Green reagent (Biorad, Cat# 172-5122). The reaction was set up as per manufacturer's instructions, using 1.5 μl of sample for each reaction. Statistical significance was performed using the two-sided Student's T-test (at α=0.05). Primers used are in Supplemental Table 1.

### Chromatin immunoprecipitation (ChIP)

1 × 10^8^ HPr-1AR cells were harvested for all ChIP assays. Cells were crosslinked in 1.0% formaldehyde (Sigma, Cat #F8775) and incubated for 10 min at RT with gentle shaking. A final concentration of 0.125 M glycine (VWR, Cat #97061-128) was added to stop the crosslinking reaction. Cells were washed twice with cold 1X PBS and then harvested in 1X PBS supplemented with protease inhibitors (Roche, Cat #11836153001). Cells were pelleted at 2,000 rpm for 4 min at 4°C. After the supernatant was aspirated, the cell pellets were lysed in 300 μl Szak's RIPA buffer (150 mM NaCl, 1.0% NP-40, 0.5% deoxycholate, 0.1% SDS, 50mM Tris-HCl pH 8.0) and incubated on ice for 10 min. Cell lysates were sonicated using a Biorupter^®^ (Diagenode, Cat #B01010002). Lysates were sonicated for 15 mins, consisting of 30 second on pulses, followed by 30 seconds resting time. Chromatin was sonicated to obtain an average smear ranging from 100-500 bps. Sonicated chromatin was spun down at 10,000 g for 10 min at 4°C. For the immunoprecipitation step, 700 μl of Szak's RIPA buffer was added to each sample to obtain a 1.0 ml lysate. Antibodies of interest (see Supplemental Table 2) were then added to each sample and incubated overnight at 4°C with slow rotation. 20 μl of protein A or G magnetic beads (Diagenode, Cat #kch-802-150, protein A; #kch-818-150, protein G) were added for 2 hrs. Beads were then washed and resuspended in 250 μl of 1.5X Talianidas Elution Buffer (70 mM Tris-HCl pH 8.0, 1 mM EDTA, 1.5% SDS) and incubated at 65°C for 10 min. The supernatant was transferred to a new tube, 13 μl of 4M NaCl was added and the samples were incubated at 65°C overnight to reverse the crosslinks. The next day, 2 μl of 2 mg/ml Proteinase K (New England Biolabs, Cat #P8102S) was added and samples were incubated at 45°C for 1 h. DNA was precipitated using PCI extraction and ethanol precipitation. ChIP DNA pellets were resuspended in 50 μl 1X TE buffer and analyzed by qRT-PCR. Data was analyzed as fold change over input. Fold change over input was calculated as (2^ΔΔCt^, where ΔΔCt = ΔCt(DHT) − ΔCt(EtOH), ΔCt = Ct(IP) − Ct(Input)). Primers used are listed in Supplementary Table 1. Antibodies used are listed in Supplementary Table 2.

### Protein extraction

HPr-1AR cells were harvested in SDS lysis buffer (50 mM Tris-HCl pH 6.8, 4.0% SDS, 10% glycerol, 5.0% β-mercaptoethanol) and sonicated for whole cell extraction. For cytoplasmic and nuclear protein separation, cells were harvested in hypotonic buffer (10 mM HEPES, 10 mM KCl, 0.1 mM EDTA, 0.1 mM EGTA, 0.4% NP-40, 50 mM NaF, 1 mM PMSF, 2 mM NaOVa, 1 mM DTT) for cytoplasmic protein extraction and in high salt buffer (20 mM HEPES, 400 mM NaCl, 1 mM EDTA, 1 mM EGTA, 50 mM NaF, 1 mM PMSF, 2 mM NaOVa, 1 mM DTT) for nuclear protein extraction. Protein concentrations were determined using the BCA assay (Pierce^®^, Cat #23227) for whole protein lysates and the Bradford Assay (Thermo Scientific, Cat #1856209) for cytoplasmic/nuclear lysates. Both assays were performed according to manufacturer's instructions.

### Western blotting

Western blotting was performed using standard protocols. PVDF membranes (Biorad, Cat #162-0177) were used for the transfer of all proteins. All primary antibodies (Supplementary Table 2) were incubated overnight at 4°C, and secondary antibodies were incubated for 1.5 h at RT. Pierce ECL western blotting substrate (Thermo Scientific, Cat #32209) was used to detect HRP-conjugated secondary antibodies via chemiluminescence and exposed to film. Exposure times ranged from seconds to hours, depending on the intensity of the signal.

### Bisulfite treatment

750 ng of DNA was bisulfite-treated with the EZ DNA Methylation Kit (Zymo Research, Cat #D5001), following the manufacturer's instructions, with the one following modification. Final bisulfite-treated DNA was eluted in 70 μl of elution buffer instead of 10 μl. Bisulfite-treated DNA was then stored at −20°C.

### Methylation analysis

Methylation analysis was performed using MassARRAY EpiTYPER analysis. This was performed as described [36]. Targeted regions for methylation analysis were amplified using specifically designed primers against androgen response elements. Primers designed are listed in the Supplemental Table 1.

### Lucia reporter assay

The 450 bp upstream ARE of SGK1 was cloned into the CpG-free lucia promoter vector (Invivogen, Cat# pcpgf-prom). As the normalizing control, the pSELECT-zeo-SEAP vector (Invivogen, Cat# psetz-seap) was used. 3 × 10^5^ cells were seeded in 6-well plates to ensure cells reach ~40% confluency in 24 h. Transfection using LipoD293 reagent (SignaGen, Cat# SL100668) was used according to the manufacturer's instructions. Briefly, using a 96-well format, 0.25 μg of plasmid (both the lucia test plasmid and the SEAP control plasmid) and 0.6 μl of LipoD293 reagent were used for each transfection, and transfections were performed in biological triplicates. To detect both secreted lucia and secreted alkaline phosphatase, Quanti-luc (Invivogen, Cat# rep-qlc1) and Quanti-Blu (Invivogen, Cat# rep-qb1) were used respectively, according to manufacturer's instructions. Lucia was measured using the Lmax luminometer machine and SEAP was measured after overnight incubation at 37°C. Statistical significance was determined using the two sided Student's T-test.

### Cloning

The *SGK1* ARE I was PCR-amplified using the primers listed in Supplemental Table 1. The primers were designed such that they carried built-in restriction enzyme cut sites for BamHI and SbfI. The PCR product was then gel-purified (Qiagen, Cat# 28706) and cloned into the pCR™2.1 vector (Invitrogen, Cat# K2040-01), according to manufacturer's instructions. One shot^®^ *E.coli* competent cells (Invitrogen, Cat# K2040-01) were used for transformation, and ampicillin (Sigma, Cat# A9393-5G) was used as the selectable marker. Restriction enzymes BamHI (New England Biolabs, Cat# R0136S) and SbfI (New England Biolabs, Cat# R0642S) were used to cut out the *SGK1* ARE I regulatory element from the pCR™2.1 vector, which was gel purified and inserted into the the CpG-free lucia promoter vector (Invivogen, Cat# pcpgf-prom), using the same reaction conditions as described above. The vector was transformed into *E.coli* GT115 chemically competent cells (Invivogen, Cat# lyo-115-11) and *E. coli* were cultured overnight at 37°C on Fast-Media^®^ Zeo agar (Invivogen, Cat# fas-zn-s).

### Plasmid preparation

Bacterial cultures were propagated overnight at 37°C in Fast-Media^®^ Zeo TB (Invivogen, Cat# fas-zn-l). Plasmid isolation was carried out using the Omega Bio-tek plasmid mini kit (Cat# D6943-02) or the Omega Bio-tek plasmid midi kit (Cat# D6904-03) according to the manufacturers' instructions.

### *In vitro* methylation

*In vitro* methylation was performed using SssI CpG methyltransferase (New England Biolabs, Cat# M0226S) for total methylation and HpaII methyltransferase (New England Biolabs, Cat# M0214S) for partial methylation. Samples were then mixed and incubated overnight at 37°C. Afterwards, each reaction was incubated at 65°C for 20 min to inactivate the enzyme. Methylated DNA was then stored at −20°C.

### Electromobility shift assay (EMSA)

EMSA was performed as previously outlined [37]. Probes were annealed in oligonucleotide annealing buffer (10 mM Tris, pH 7.5-8.0, 50 nM NaCl and 1 mM EDTA). Equal volumes of complementary oligos were mixed together and incubated at 95°C for 5 min. The mixture was then slowly cooled down to 37°C.

## SUPPLEMENTARY MATERIAL FIGURES


