# Supplementary material for: Hormone stimulation of androgen receptor mediates dynamic changes in DNA methylation patterns at regulatory elements
Source: Oncotarget. 2015 Dec 4;6(40):42575–89. doi: 10.18632/oncotarget.6471 (PMC4767454; doi:10.18632/oncotarget.6471)
Supplement: Supplementary file 1 [file oncotarget-06-42575-s001.pdf]

Hormone stimulation of androgen receptor mediates dynamic changes in DNA methylation patterns at regulatory elements

Supplementary Material

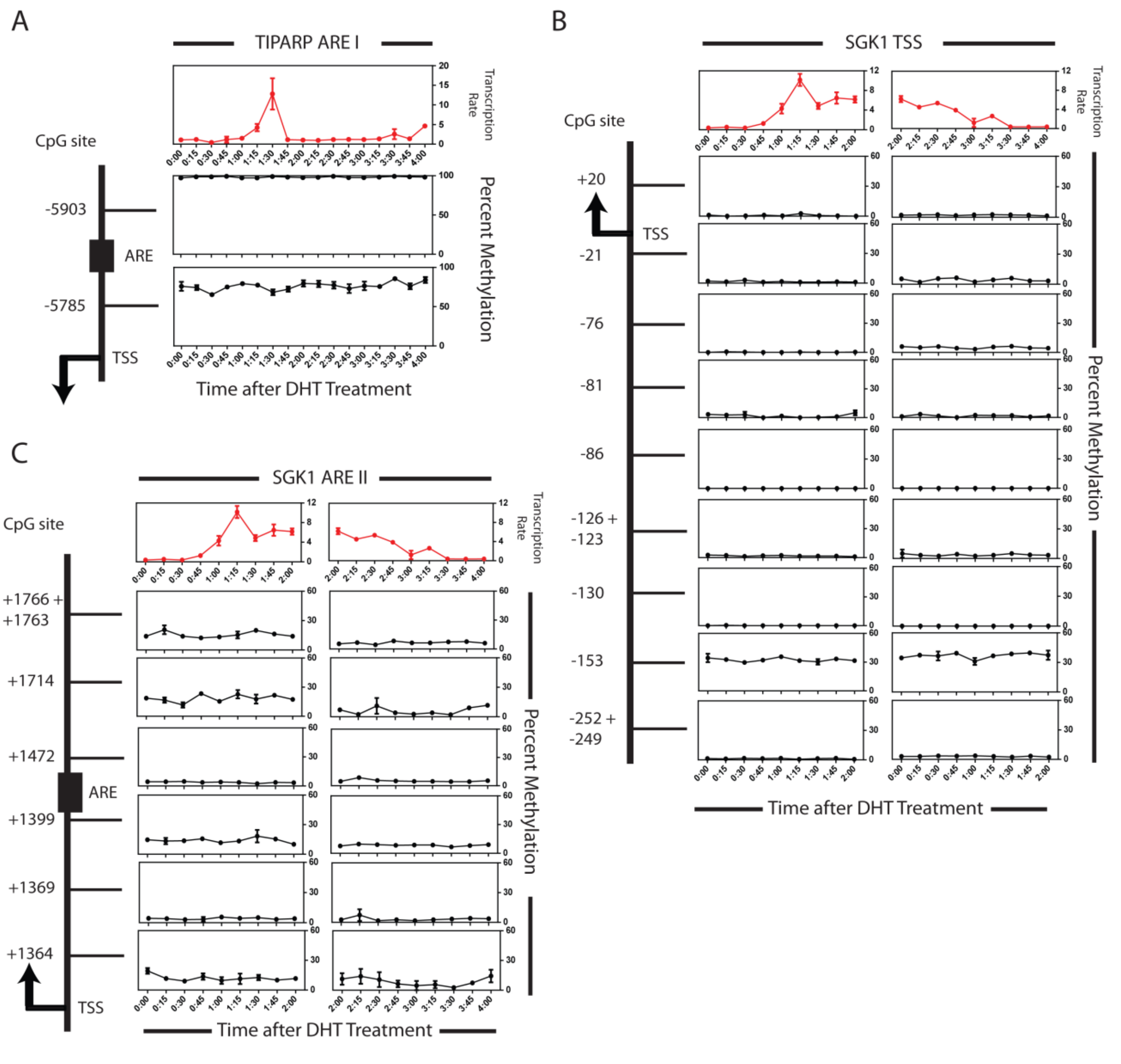

Supplemental Figure 1A-C: No methylation dynamics observed at other AREs.

HPr-1AR cells were treated with 10nM DHT and harvested for RNA and DNA, from the same cell pellet, at the indicated time points. Transcription data is reflective of normalized quantity of nascent gene expression. Expression data was normalized to β-microglobulin and representative of three biological replicates. CpG sites in the promoter region of *SGK1* were interrogated for methylation. Each line represents a single CpG site, where each CpG site is labeled in respect to

its distance in base pairs from the TSS (as noted by the arrow). The black box represents the confirmed androgen response element (ARE) from previously published ChIP-chip studies.[24] All methylation data points are representative of three biological replicates or best two out of three replicates.

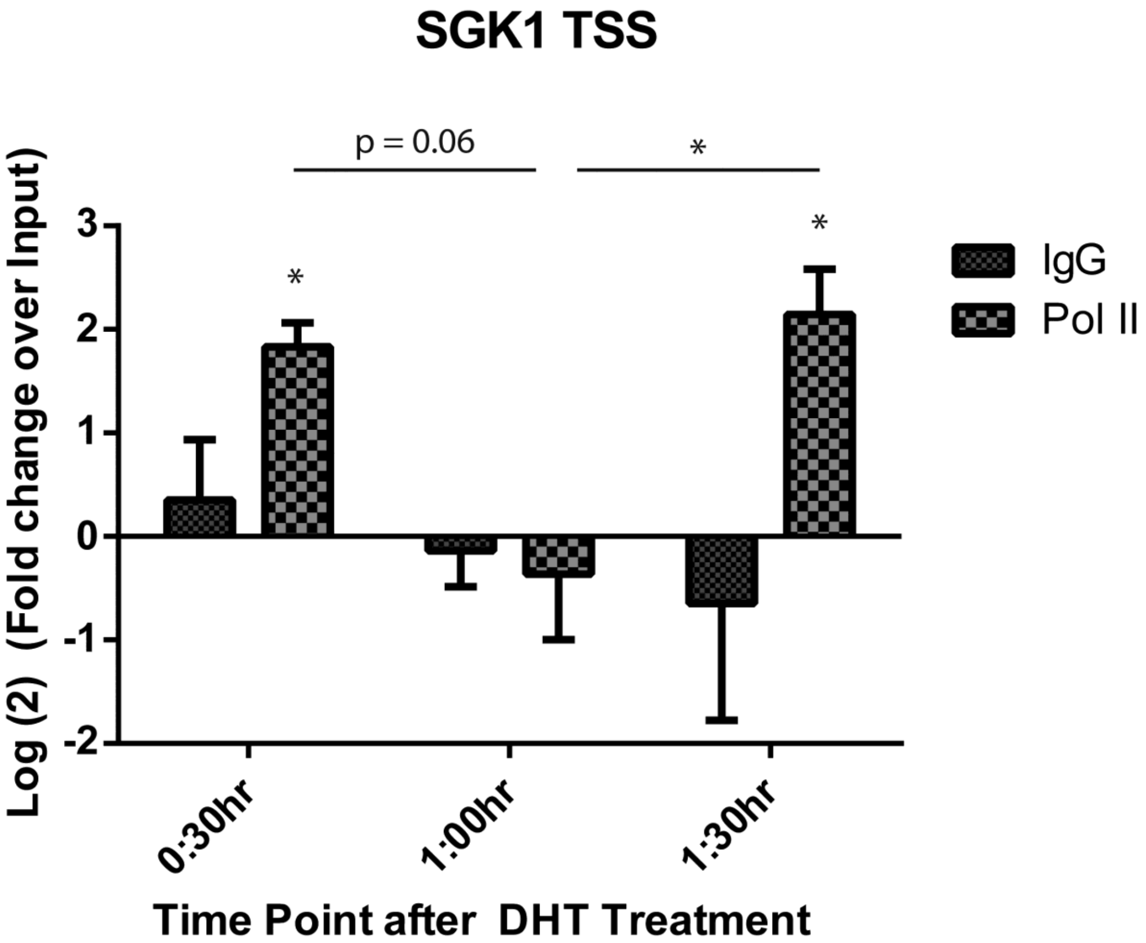

Supplemental Figure 2: Dynamic recruitment of RNA Pol II.

HPr-1AR cells were treated with EtOH vehicle or 10 nM DHT for indicated time points. Promoter occupancy was determined using antibodies against RNA Polymerase II and normalized and presented as fold change over input. ChIP signal was measured using qRT-PCR with primers specific to the interrogated region. All data points are representative of biological triplicates. (Student T-test, $*p < 0.05$).
